# Critical roles of the *ddx5* gene in zebrafish sex differentiation and oocyte maturation

**DOI:** 10.1038/s41598-020-71143-2

**Published:** 2020-09-01

**Authors:** Ryota Sone, Kiyohito Taimatsu, Rie Ohga, Toshiya Nishimura, Minoru Tanaka, Atsuo Kawahara

**Affiliations:** 1 Laboratory for Developmental Biology, Center for Medical Education and Sciences, Graduate School of Medical Science, University of Yamanashi, 1110 Shimokato, Chuo, Yamanashi 409-3898 Japan; 2 Division of Biological Science, Graduate School of Science, Nagoya University, Nagoya, 464-8602 Japan; 3 Present Address: Faculty of Fisheries Science, Hokkaido University, Sapporo, 041-8611 Japan

**Keywords:** Developmental biology, Germline development, Oogenesis

## Abstract

DEAD-box helicase 5 (Ddx5) functions as an ATP-dependent RNA helicase and as a transcriptional coactivator for several transcription factors; however, the developmental function of the *ddx5* gene in vertebrates is not fully understood. We found that the zebrafish *ddx5* gene was expressed in developing gonads. Using the genome editing technology transcription activator-like effector nuclease, we established a *ddx5*-disrupted zebrafish and examined the morphological phenotypes of the mutant. We found that the majority of *ddx5*-deficient mutants developed as fertile males with normal testes and a small number of *ddx5*-deficient mutants developed as infertile females with small ovaries. Apoptotic cell death at 31 days post fertilization was increased in thick immature gonads (presumptive developing ovaries) of the *ddx5*-deficient mutant compared to those of heterozygous wild-type fish, while the number of apoptotic cells in thin immature gonads (presumptive developing testes) was comparable between the mutant and wild-type animals. Histological analysis revealed that ovaries of adult *ddx5*-deficient females had fewer vitellogenic oocytes and a larger number of stage I and II oocytes. The amount of cyclic adenosine monophosphate in the *ddx5*-deficient ovaries was high compared to that of wild-type ovaries, presumably leading to the mitotic arrest of oocyte maturation. Therefore, the *ddx5* gene is dispensable for testis development, but it is essential for female sex differentiation and oocyte maturation in zebrafish.

## Introduction

Vertebrates exhibit various types of sex determination system, leading to the differentiation of gonads into testis or ovary^1^. Recent findings have suggested that zebrafish possess a ZZ/ZW sex determination system^2,3^. However, such a system was lost in most laboratory zebrafish stocks^4^, presumably utilizing a polygenic sex determination system. Furthermore, various environmental factors, including population density and temperature, can influence the sex proportion in zebrafish^5,6^. Immature gonads during the sex differentiation period start to differentiate as bipotential juvenile ovaries. Immature gonads in approximately half of the zebrafish population develop into ovaries around 30 days post fertilization (dpf), while the immature gonads in the remaining population develop into testes^7^. Thus, the degree of apoptosis in immature gonads during the sex differentiation period may be essential for testicular and ovarian differentiation in zebrafish. However, it is not fully understood what kind of genes are involved in sex differentiation in zebrafish.

The DEAD-box helicase (Ddx) family is defined by a conserved DEAD (Asp-Glu-Ala-Asp) motif involved in ATP hydrolysis, and family members also possess several conserved motifs that have in ATPase and helicase activity^8^. DEAD-box helicases play important roles in various cellular processes, such as the regulation of transcription, RNA processing and ribosome biogenesis^9^. It is well known that *vasa/ddx4* is expressed in the germ cells of organisms from fruit flies to mammals^10,11^. The *vasa/ddx4*-disrupted zebrafish developed exclusively as infertile males^12^, indicating a critical role in gametogenesis. The human *DDX5* gene is expressed in spermatogonia^13^. The ablation of the *Ddx5* gene using tamoxifen-inducible *Ddx5* knockout male mice results in the rapid loss of spermatogonia^14^. Thus, the physiological function of *ddx5* in other vertebrates is not known.

We found that the zebrafish *ddx5* gene was expressed in developing gonads. To examine the loss of function of the *ddx5* gene, we disrupted the *ddx5* gene in zebrafish with TALEN. The majority of *ddx5*-deficient mutants developed as fertile males, while a small population of *ddx5*-deficient mutants developed as infertile females with small ovaries. Such phenotypes are different from those of *vasa/ddx4*-disrupted zebrafish and *Ddx5*-disrupted male mice^12,14^. We found that apoptotic cell death was increased in thick immature gonads (presumptive developing ovaries) of *ddx5*-deficient mutants at 31 dpf. Furthermore, ovaries of adult *ddx5*-deficient females predominantly possessed stage I and II oocytes and maintained high cyclic adenosine monophosphate (cAMP) concentrations. These results suggest that the *ddx5* gene is essential for sex differentiation and oocyte maturation in zebrafish.

## Results

### Developmental expression of *ddx5* during zebrafish gonad development

**Figure 1:**
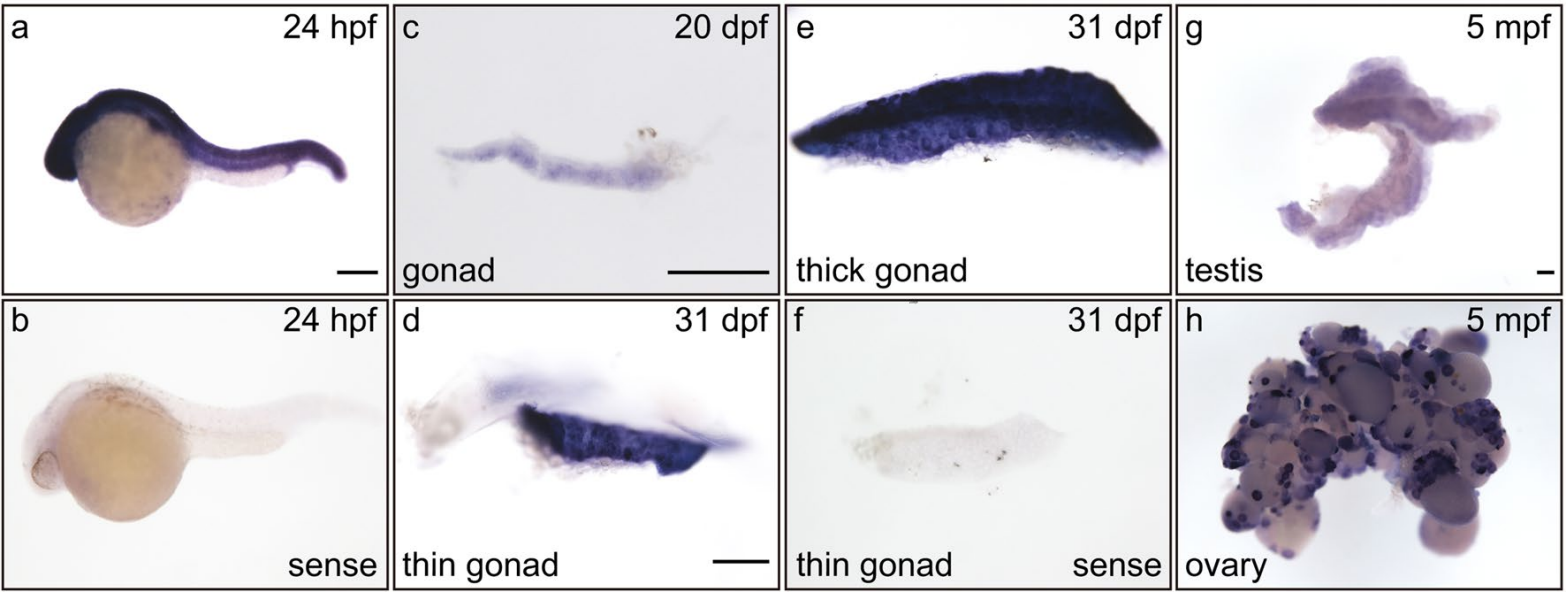
Developmental expression of the *ddx5* gene in zebrafish gonads. The expression of *ddx5* was examined by whole-mount in situ hybridization (WISH) using sense *ddx5* RNA probes (**b**, **f**) and antisense *ddx5* RNA probes (**a**, **c**–**e**, **g**, **h**). (**a**, **b**) 24 hpf embryos. (**c**) 20 dpf immature gonad. (**d**, **f**) 31 dpf thin immature gonads. (**e**) 31 dpf thick immature gonad. (**g**) 5 mpf testis. (**h**) 5 mpf ovary. *ddx5* expression was widely detected in 24 hpf embryos (**a**) and weakly detected in 20 dpf immature gonads (**c**). The *ddx5* gene was strongly expressed in thin and thick immature gonads at 31 dpf (**d**, **e**). The *ddx5* expression is strongly detected in stage I and II oocytes (**h**), while the *ddx5* gene is weakly expressed in whole testes at 5 mpf (**g**). Scale bar 200 μm.

Recent accumulating evidence shows that some of *Ddx* family genes are expressed during gonad development^15,16^. To examine the developmental expression of the *ddx5* gene, we performed whole-mount in situ hybridization (WISH) using several developmental stages of zebrafish embryos and gonads. The *ddx5* gene was widely expressed in embryos at 24 h post fertilization (hpf) (Fig. 1a,b). *ddx5* expression at 20 dpf was weakly detected in immature gonads (Fig. 1c) and strongly detected in thin and thick immature gonads at 31 dpf, which presumably progress towards initial testicular and ovarian differentiation, respectively (Fig. 1d–f). The *ddx5* gene was significantly expressed in stage I and II oocytes of adult ovaries, while *ddx5* expression was weakly detected in whole testes at 5 months post fertilization (mpf) (Fig. 1g,h). Thus, these results indicate that the *ddx5* gene is predominantly expressed in the immature gonads at 31 dpf and in the early differentiation stages of the ovary at 5 mpf.

### Sex distribution and fertility of *ddx5*-deficient zebrafish

**Figure 2:**
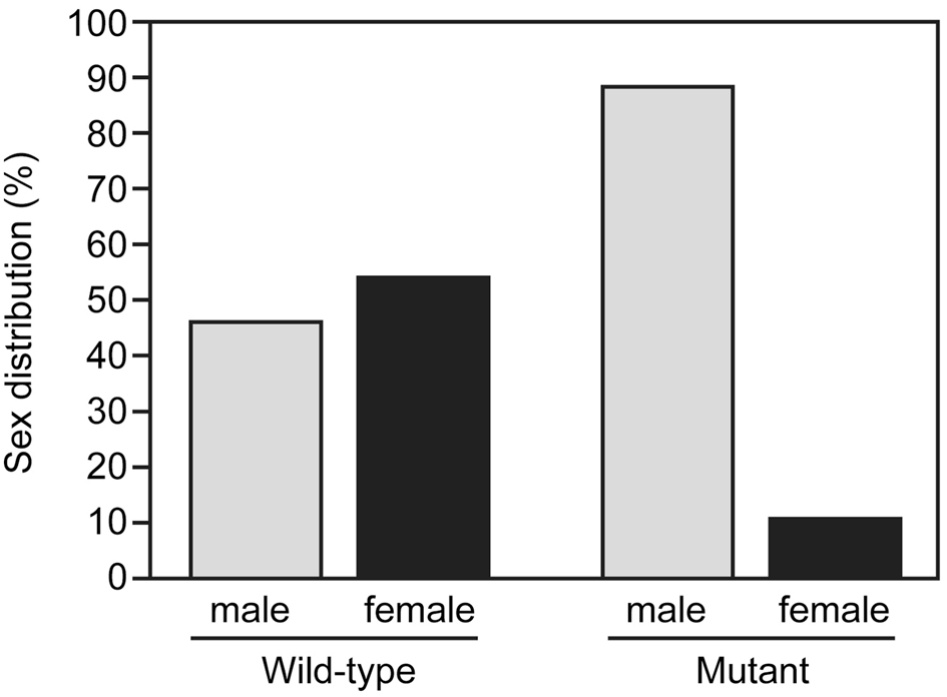
Disruption of the *ddx5*^−/−^ gene causes abnormal sex ratio. Homozygous *ddx5*^−/−^ male mutants (5 mpf) crossed heterozygous *ddx5*^+/−^ females (5 mpf), and the sex of growing adult fish was determined by anatomical features (testis or ovary). Total number of progeny: n = 199. Genomic DNA was prepared from individual caudal fins, and the genotype of each fish was determined by genomic PCR using the *ddx5* locus-specific primers. The number of heterozygous wild-type animals: n = 112. The number of homozygous mutant animals: n = 87.

To establish the *ddx5*-disrupted zebrafish line, *ddx5*-TALEN constructs were injected into one-cell stage zebrafish embryo, and the F0 embryos were raised to adulthood. The *ddx5* mutant allele *uy210*, which had a total deletion of 5 base pairs (bp), was isolated (***Supplemental Fig. S1–Fig. S3). The Ddx5 mutant protein was functionally disrupted because the mutant did not possess most functional domains, such as the Q motif, helicase ATP-binding domain and transactivation domain (Supplemental Fig. S1). We could not observe any apparent embryonic abnormality in the *ddx5*-deficient embryos at 5 dpf (Supplemental Fig. S4). Because *ddx5* expression is detected in immature gonads at 31 dpf and in adult oocytes and testes at 5 mpf, we examined the sex distribution and fertility in *ddx5*-deficient adult fish. Fertility was determined by mating individual *ddx5*-deficient fish with three mature wild-type fish. The *ddx5*-deficient males were fertile, whereas the *ddx5*-deficient females were infertile (Table 1). We determined the genotype of adult fish and examined the gonad morphology of the progeny of the *ddx5*^−/−^ males and *ddx5*^+/−^ females cross. The homozygous *ddx5*^−/−^ fish at 5 mpf were predominantly males with testes (77 males and 10 females), while the sex ratio of heterozygous *ddx5*^+/−^ fish containing the wild-type allele was almost evenly balanced (52 males and 60 females) (Fig. 2). These results suggest that the *ddx5* gene is involved in sex differentiation and ovarian development. Therefore, we focused on the loss of function analysis of the *ddx5* gene in immature gonads and during oocyte maturation.

**Table 1:** Fertilization ability of the *ddx5*^*−/−*^ mutant.

Male	Female	The proportion of successful fertilization in three trials
*ddx5*^*−/−*^ ♂ No. 1	WT ♀ (n = 3)	3/3
*ddx5*^*−/−*^ ♂ No. 2	WT ♀ (n = 3)	3/3
*ddx5*^*−/−*^ ♂ No. 3	WT ♀ (n = 3)	3/3
*ddx5*^*−/−*^ ♂ No. 4	WT ♀ (n = 3)	3/3
WT ♂ (n = 3)	*ddx5*^*−/−*^ ♀ No. 5	0/3
WT ♂ (n = 3)	*ddx5*^*−/−*^ ♀ No. 6	0/3
WT ♂ (n = 3)	*ddx5*^*−/−*^ ♀ No. 7	0/3
WT ♂ (n = 3)	*ddx5*^*−/−*^ ♀ No. 8	0/3

An individual *ddx5*^*−/−*^ male (No. 1–No. 4) or female mutant (No. 5–No. 8) was mated three times with three wild-type fish.

### Proliferating and dying cells in the immature gonads of *ddx5*-deficient zebrafish

**Figure 3:**
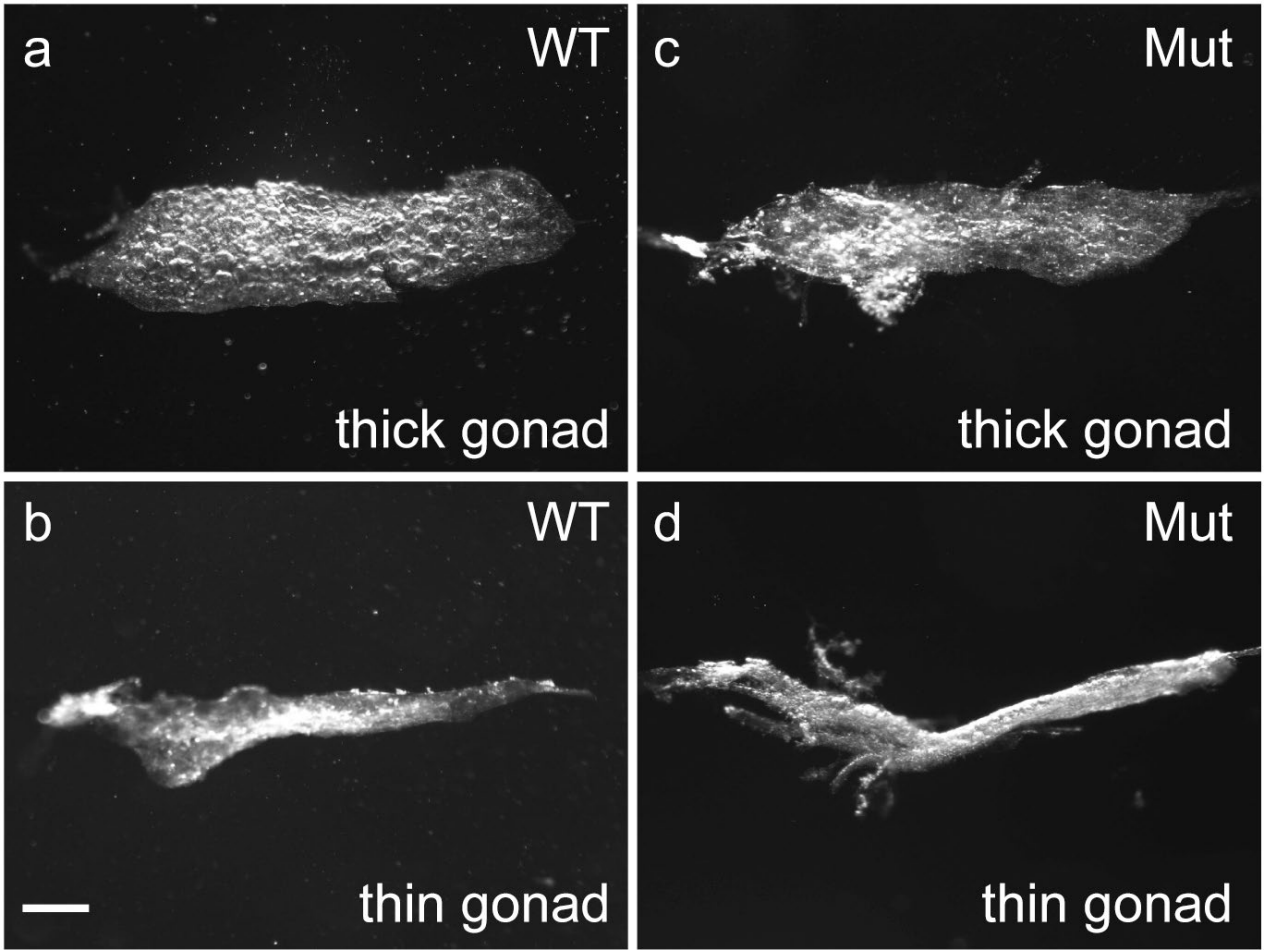
Morphology of immature gonads in the *ddx5*-deficient fish at 31 dpf. (**a**) Wild-type, thick immature gonad. (**b**) Wild-type, thin immature gonad. (**c**) *ddx5*^*−/−*^ mutant, thick immature gonad. (**d**) *ddx5*^*−/−*^ mutant, thin immature gonad. Scale bar 200 μm.

The maintenance of a sufficient number of germ cells is required for female sex determination in zebrafish. Thus, apoptosis is involved in zebrafish sex differentiation^17^. We observed thick immature gonads (presumptive developing ovaries) and thin immature gonads (presumptive developing testes) in wild-type fish at 31 dpf (Fig. 3). In the *ddx5*-deficient gonads, there were thick immature gonads and thin immature gonads at 31 dpf. Thus, the immature gonads in wild-type and the mutant at 31 dpf are similar in appearance.

**Figure 4:**
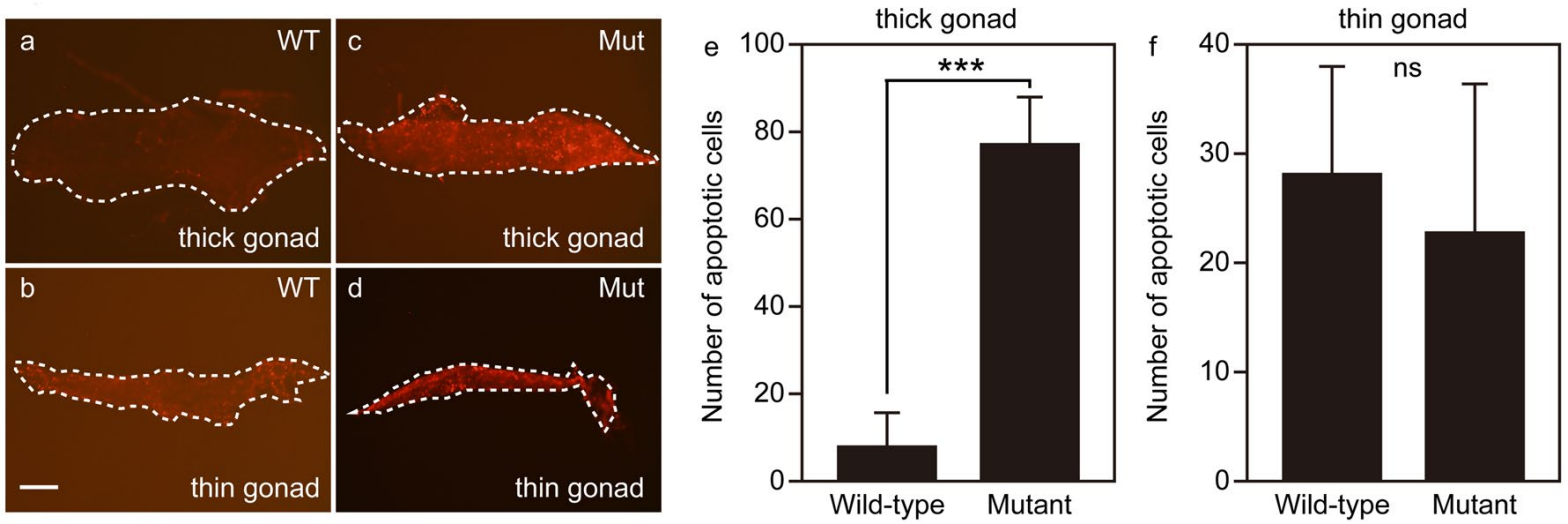
Dying cells in immature gonads of *ddx5*-deficient fish. (**a**–**f**) TUNEL analysis. (**a**, **b**) Wild-type. (**c**, **d**) *ddx5*^−/−^ mutant. (**a**, **c**, **e**) Thick immature gonads. (**b**, **d**, **f**) Thin immature gonads. Scale bar 200 μm. (**e**) The number of apoptotic cells in wild-type (**e**; n = 12, total 94 positive cells) (**f**; n = 4, total 113 positive cells) and *ddx5*^*−/−*^ mutants (**e**; n = 7, total 538 positive cells) (**f**; n = 7, total 160 positive cells) were counted. Error bars indicate standard deviation. Asterisks indicate statistical significance between wild-type and mutant samples (by t test). ***P < 0.001. *ns* not significant.

We examined the number of proliferating and dying cells in immature gonads of *ddx5*-deficient fish and wild-type fish at 31 dpf. TUNEL analysis revealed that apoptotic cells increased in number in thick immature gonads of *ddx5*-deficient fish compared to those of wild-type fish (Fig. 4), while the number of apoptotic cells between wild-type and *ddx5*-deficient thin immature gonads was comparable. The number of proliferating cells marked by anti-phosphorylated histone H3 immunostaining was comparable in the mutant and wild-type immature gonads (Supplemental Fig. S5).

### *ddx5*-deficient ovaries exhibited oocyte maturation defects and maintained high cAMP concentrations

**Figure 5:**
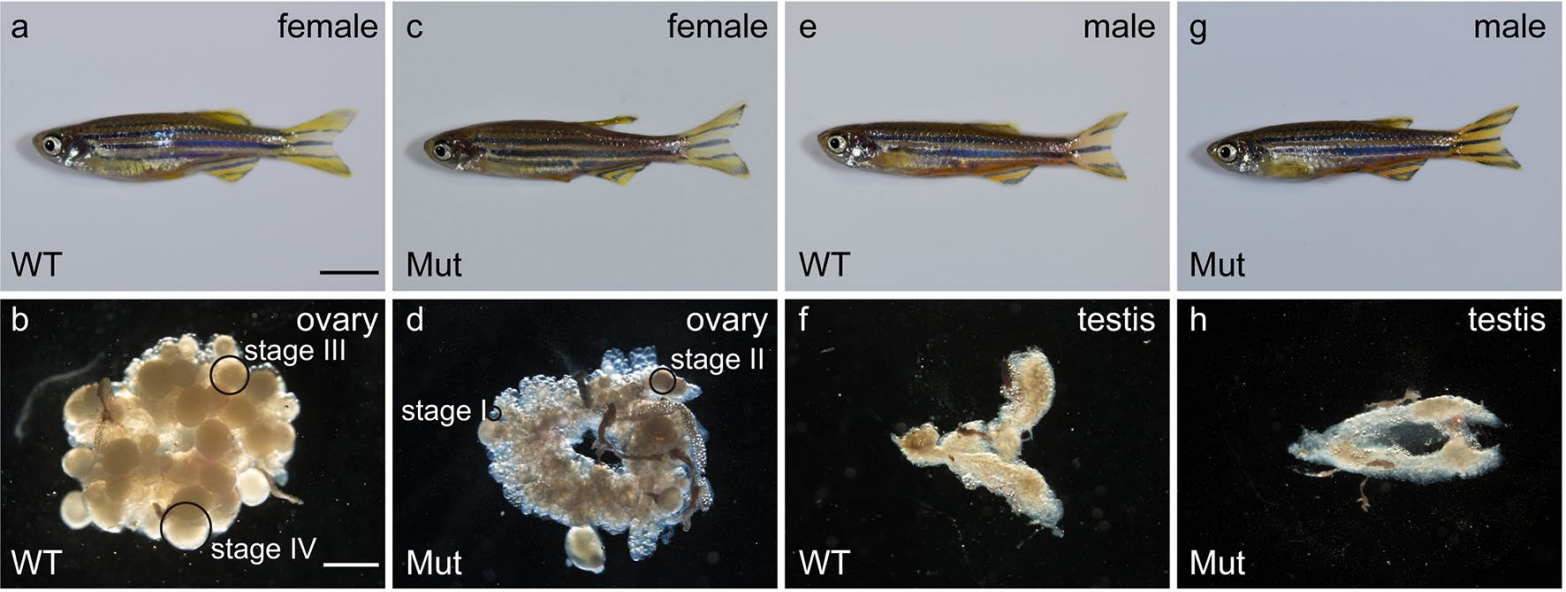
Homozygous adult *ddx5*^*−/−*^ females had small ovaries containing immature oocytes. (**a**, **b**, **e**, **f**) *ddx5*^+*/−*^ wild-type at 5 mpf. (**c**, **d**, **g**, **h**) The *ddx5*^*−/−*^ mutants at 5 mpf. The ovaries of *ddx5*-deficient females predominantly possessed stage I and II embryos, while various ovarian stages, from I to IV, were observed in wild-type females. The testes of wild-type and *ddx5*-deficient fish were similar. Genotyping of individual fish was performed by genomic PCR. (**a**) Scale bar 5 mm. (**b**) Scale bar 1 mm.

**Figure 6:**
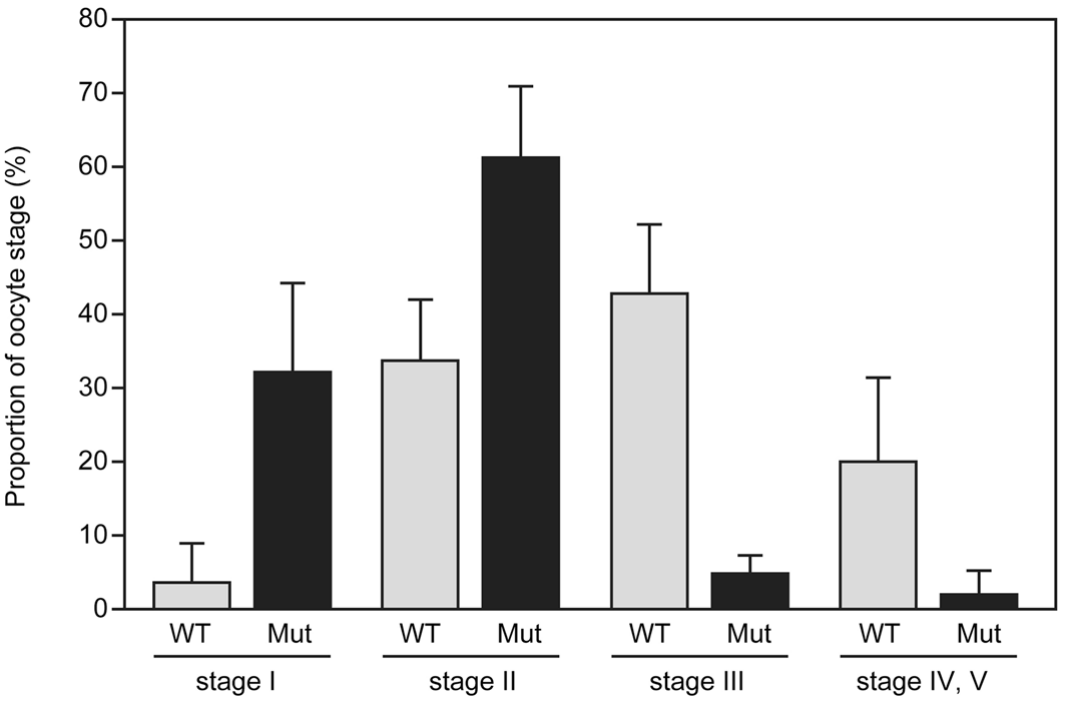
Distribution of the *ddx5*-deficient oocytes classified by oocyte maturation. Ovarian follicles from wild-type (n = 5) and mutant animals (n = 5) at 5 mpf were classified by diameter (stage I; 7–140 μm, stage II; 140–340 μm, stage III; 340–690 μm, stage IV, V; 690–750 μm). Fifty ovarian follicles from individual ovaries represent the proportion of oocyte stages. Error bars indicate standard deviation.

**Figure 7:**
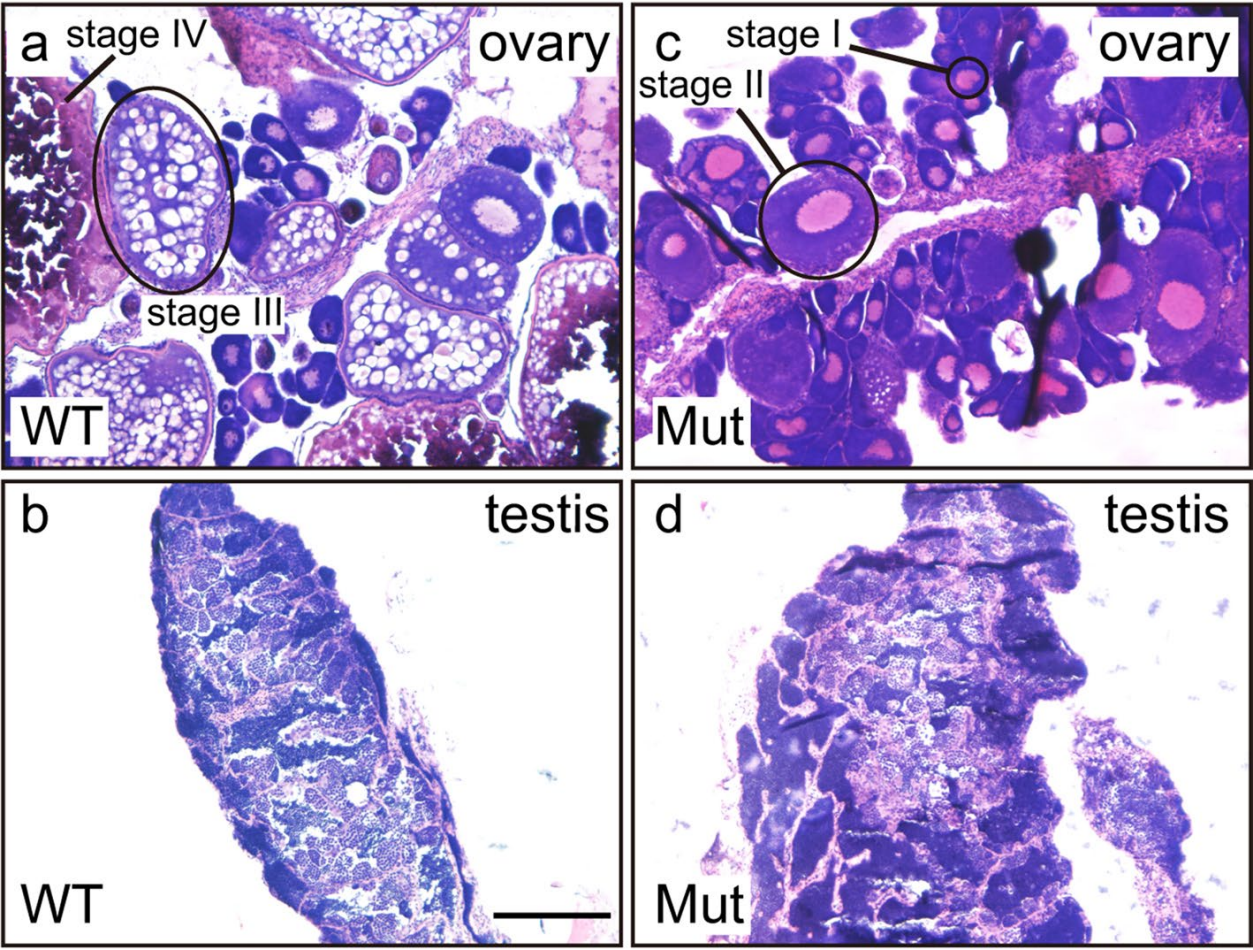
Hematoxylin and eosin (HE) staining of gonadal sections. (**a**, **b**) *ddx5*^+*/−*^ wild-type gonads. (**c**, **d**) the *ddx5*^*−/−*^ mutant gonads. (**a**, **c**) Wild-type ovaries possessed stage I–IV oocytes, whereas *ddx5*-deficient ovaries predominantly contained stage I and II oocytes. (**b**, **d**) Both wild-type and *ddx5*-deficient testes contained differentiating germ cells. Scale bar 200 μm.

Morphologies of *ddx5*-deficient gonads were examined. We found that the *ddx5*-deficient female fish had small ovaries at 90 dpf and 5 mpf (Fig. 5, Supplemental Fig. S6). Wild-type ovaries contained various stages^18^, and most were in intermediate stages II and III, while few were in early stage I and late stage IV–V; however, the ovaries of *ddx5*-deficient females possessed high amounts of stage I and II oocytes and few stage III–V oocytes (Fig. 6). Distribution of oocyte stages was different between wild-type and *ddx5*-mutant (P < 0.001 for both wild-type and *ddx5*-deficient oocytes; Chi square test). In contrast, the testes of *ddx5*-deficient males had morphology that was similar to that of wild-type males (Fig. 5). Histological analysis confirmed that the ovaries of *ddx5*-deficient fish predominantly possessed stage I and II oocytes and few stage III-V oocytes, while the testes containing sperm were comparable between the mutant and wild-type fish (Fig. 7).

**Figure 8:**
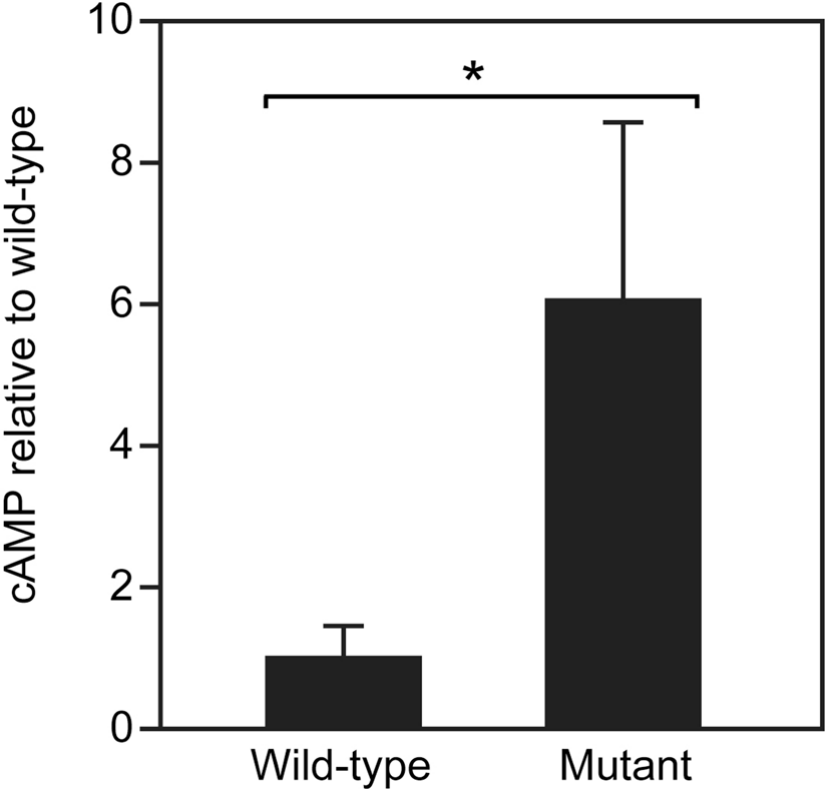
cAMP concentration relative to the wild-type ovaries. The concentration of cAMP in each ovary (wild-type: n = 4, *ddx5*^*−/−*^ mutant: n = 4,) was determined with a cAMP ELISA kit. Error bars indicate standard deviation. Asterisks indicate statistical significance between wild-type and the mutant levels (by t test). *P < 0.05.

A high concentration of cyclic AMP (cAMP) in fish oocytes is required for maintaining meiotic arrest^19,20^. We examined the cAMP concentration in wild-type and *ddx5*-deficient ovaries. The concentration of cAMP was higher in *ddx5*-deficient ovaries than it was in wild-type ovaries (Fig. 8). These results suggest that high amounts of stage I and II oocytes are present due to mitotic arrest that was mediated by a maintained high concentration of cAMP.

## Discussion

In this study, we first reported the loss of function of the *ddx5* gene in zebrafish. We have demonstrated that the majority of *ddx5*-deficient mutants develop as fertile males with normal testes, whereas a few *ddx5*-deficient fish develop into infertile females with aberrant small ovaries (Figs. 2, 5; Table 1). This phenotype was quite different from the failure of *Ddx5*-disrupted male mice on spermatogenesis^14^.

The expression of *ddx5* was widespread in whole embryos and was not specifically detected in the primordial germ cells at 24 hpf (Fig. 1). *ddx5* expression at 24 hpf was different from the germ cell-specific expression of the *vasa/ddx4* and *nanos1* genes^21,22^. We found that the *ddx5* gene was expressed in developing gonads at 20 dpf. Although *ddx5* expression was detected in thin immature gonads (presumptive developing testes) and thick immature gonads (presumptive developing ovaries) at 31 dpf, it was found to have much stronger expression in adult ovaries compared to adult testes. Therefore, *ddx5* expression gradually decreased in the developing testis compared to the developing ovary, suggesting important roles of the *ddx5* gene in ovarian development.

Recent studies have shown that a threshold number of immature gonads is required for the progression of ovarian fate in zebrafish and mice^17,23,24^. Induction of apoptosis in the gonad is important for testis development. In fact, Fancl is a member of the Fanconi anemia/BRCA DNA repair pathway, and homozygous zebrafish *fancl* mutants exclusively develop as fertile males^25^. The abnormal sex ratio phenotype in the *fancl* mutant is caused by abnormally increased apoptosis in immature gonads. We observed that most *ddx5*-deficient mutants developed as fertile males (Fig. 2). We found that thick immature gonads (presumably developing ovaries) in *ddx5*-deficient fish were accompanied by an abnormal increase in apoptosis compared to wild-type fish during the sex differentiation period (Fig. 4). Therefore, increased apoptosis in the developing gonad provides a cellular mechanism for abnormal sex ratio phenotype of *ddx5*-deficient zebrafish.

During gonad maturation stages, the *ddx5*-deficient mutant had morphologically similar testis (Fig. 5) and fertilization activity (Table 1). In clear contrast, the ovaries of *ddx5*-deficient fish possessed an abundance of stage I and II oocytes, raising the possibility of mitotic arrest during oocyte maturation. It is not clear why a small number of gonads can develop the ovaries. Histological analysis confirmed that the ovaries of adult *ddx5*-deficient females had fewer vitellogenic oocytes and a high number of stage I and II stage oocytes (Fig. 7). Thus, the *ddx5* gene is not required for initial oocyte maturation, but is necessary for vitellogenic oocytes. In most of fish oocytes, elevated intraoocyte cAMP maintains protein kinase A (PKA) in an active state that ascertains cell cycle arrest^19^. Thus, a decrease in cAMP concentration in oocytes is necessary for the resumption of meiosis. The concentration of cAMP was high in *ddx5*-deficient ovaries compared to wild-type ovaries (Fig. 8). A high concentration of cAMP contributes to the abundance of stage I and II oocytes. Another possibility is that the loss of the *ddx5* gene causes oocyte arrest unrelated to the regulation of cAMP levels and oocyte arrest in the *ddx5*-deficient mutant results in high cAMP concentration. In summary, the *ddx5* gene is dispensable for testis development but indispensable for oocyte maturation in zebrafish.

## Methods

### Whole-mount in situ hybridization (WISH)

The accession number of zebrafish *ddx5* gene is LC565489. The expression of *ddx5* was examined by WISH as previously described^26^. Zebrafish embryos and gonads were hybridized with the digoxygenin (DIG)-labelled RNA probe at room temperature for overnight. After three time washing with PBS containing 0.1% Tween-20 (PBST), the samples were incubated with alkaline phosphatase-conjugated anti-DIG antibody. After three time washing with PBST, the samples were incubated with BM Purple (Roche) as the substrate to visualize the RNA probe recognized by the anti-DIG antibody. After three time washing with PBST, the samples were fixed in 4% paraformaldehyde.

### Construction of TALEN plasmids and microinjection of TALEN mRNA

The plasmids for synthesizing TALEN mRNAs were constructed with a two-step assembly system, as described previously^27^. Initially, six or fewer TAL effector repeat modules were ligated into pFUS vectors. Intermediate array vectors and last TAL effector repeat were then ligated into a pCS2TAL3DDD vector to generate a forward TALEN or a pCS2TAL3RRR vector to generate a reverse TALEN^28^. The amino acid sequences of the constructed TALENs for *ddx5* are shown in Supplementary Table S2.

The plasmids used for synthesizing TALEN mRNAs were linearized by NotI digestion, and mRNAs were transcribed using a mMESSAGE mMACHINE SP6 kit (Life Technologies) and purified using an RNeasy Mini Kit (QIAGEN). Forward and reverse TALEN mRNAs (400 pg each) were simultaneously injected into zebrafish blastomeres at the one-cell stage of embryonic development.

### Genotyping for the *ddx5* locus and genomic sequencing

To prepare genomic DNA, the embryos and tissues at the indicated stages were incubated in 108 μl of 50 mM NaOH at 98 °C for 10 min. Subsequently, 12 μl of 1 M Tris–HCl (pH 8.0) was added to the solution^29^. Genomic fragments at the targeted sites were amplified by PCR with PrimeTaq (Primetech), and the locus-specific primers are listed in Supplementary Table S2. PCR conditions were as follows: 40 cycles of 98 °C for 10 s, 55 °C for 30 s and 72 °C for 30 s. To perform the heteroduplex mobility assay (HMA), the resultant PCR amplicons were electrophoresed on a 12.5% polyacrylamide gel^29^. To confirm individual mutations, genomic fragments for the targeted genomic locus were amplified from 1 μl of the solution using PCR (Supplementary Table S2). The resultant PCR fragments were subcloned into the pGEM-T Easy vector (Promega) and genomic sequences were determined by sequence analysis.

### Histological analysis

Embryos were dehydrated in 80% ethanol and embedded using a Technovit kit (Kulzer)^30^. Embedded embryos were sectioned on a Leica RM2125 microtome at 7 μm and mounted on slides. Embryos were stained with hematoxylin–eosin (HE) after sectioning.

### Detection of dying and proliferating cells

To detect proliferating cells, gonads at 31 dpf were incubated with anti-phospho-histone H3 antibody (1/500 dilution) (Upstate, #06-570) in PBST containing sheep serum (10%) at 28 °C overnighty^31^. After three times washes with PBST, the embryos were incubated with Alexa Fluor 594 goat anti-rabbit IgG (1/500 dilution) (Invitrogen) in PBST containing sheep serum (10%) at 25 °C for 4 h. After three times washes with PBST, the proliferating cells were observed by fluorescence stereomicroscopy. We counted the red, rounded signals as proliferating cells and the other signals as negative cells.

Fixed gonads were dehydrated and treated with proteinase K (10 ng/μl) in PBST for 5 min. After three times washes with PBST, the gonads were incubated with TdT reaction cocktail (400 μl/sample) (Invitrogen) for 60 min at 37 °C. Then they were added to Click-iT reaction cocktail (400 μl/sample), where they were incubated for 30 min at 25 °C, in the dark. Apoptotic cells labelled with Alexa Fluor 594 were observed by fluorescence stereomicroscopy. We counted the red, rounded signals as apoptotic cells and the other signals as negative cells.

### Measurement of cAMP concentration

Cell extract was prepared from 5 mpf ovaries of wild-type and *ddx5*-deficient fish. The concentration of cAMP in each sample was analyzed with a cAMP ELISA kit (Caymen Chemical Company) following the manufacturer’s instructions.

### Ethics statement

All animal experiments were performed in accordance with the animal protocol approved by the Institutional Animal Care and Use Committee (IACUC) and the ethics committee of the University of Yamanashi. The IACUC and the ethics committee of the University of Yamanashi approved this study (Approval Identification Number: A30-25).

## Supplementary information

Supplementary file1
