# Supplementary material for: Critical roles of the ddx5 gene in zebrafish sex differentiation and oocyte maturation
Source: Sci Rep. 2020 Sep 1;10:14157. doi: 10.1038/s41598-020-71143-2 (PMC7463030; doi:10.1038/s41598-020-71143-2)
Supplement: Supplementary file 1 — Supplementary file1 [file 41598_2020_71143_MOESM1_ESM.docx]

**Critical roles of the *ddx5* gene in zebrafish sex differentiation and oocyte maturation**

Ryota Sone^1^, Kiyohito Taimatsu^1^, Rie Ohga^1^, Toshiya Nishimura^2^, Minoru Tanaka^2^, and Atsuo Kawahara^1^

^1^Laboratory for Developmental Biology, Center for Medical Education and Sciences, Graduate School of Medical Science, University of Yamanashi, 1110 Shimokato, Chuo, Yamanashi, 409-3898, Japan

^2^Division of Biological Science, Graduate School of Science, Nagoya University, Nagoya, 464-8602, Japan

Correspondence and requests for materials should be addressed to A.K. (e-mail: akawahara@yamanashi.ac.jp)

**Supplemental Information**

**Supplemental Figures**


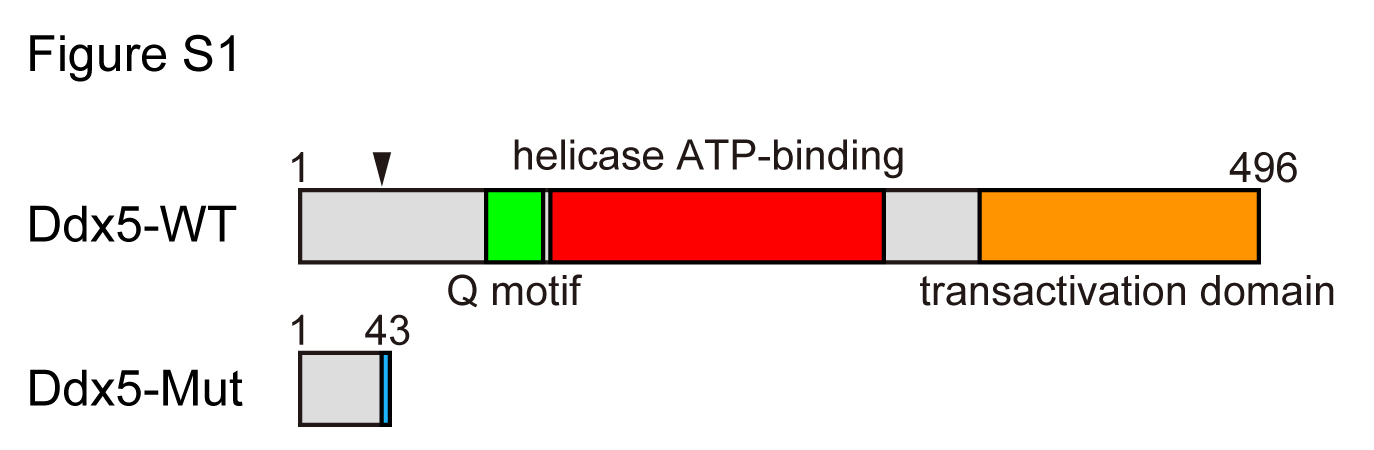


**Figure S1. Molecular structure of wild-type and mutant Ddx5 proteins.** The *ddx5^-/-^* lesions cause premature stop codons after 6 missense amino acids (blue rectangle) starting at amino acid 43. The arrowhead indicates the TALEN target site. The Q motif in Ddx5 is indicated in a green rectangle. The helicase ATP-binding domain in Ddx5 is indicated by a red rectangle. The transactivation domain in Ddx5 is indicated by an orange rectangle.

Figure S2

Ddx5 Wild-type

MPGYSDRDRGRDRGSYSSGPPRFGGSRNGPPPAKKFGNPGDRLRKKHWNLDELPKFEKNFYQENPDVARRSAQEVEHYRRSKEITVKGRDGPKPIVKFHEANFPKYVMDVITKQNWTDPTPIQAQGWPVALSGKDMVGIAQTGSGKTLSYLLPAIVHINHQPFLEHGDGPICLVLAPTRELAQQVQQVAAEYGKASRIKSTCIYGGAPKGPQIRDLERGVEICIATPGRLIDFLEAGKTNLRRCTYLVLDEADRMLDMGFEPQIRKIVDQIRPDRQTLMWSATWPKEVRQLAEDFLKEYIQINVGALQLSANHNILQIVDVCNDGEKEDKLIRLLEEIMSEKENKTIIFVETKRRCDDLTRRMRRDGWPAMGIHGDKNQQERDWVLNEFKYGKAPILIATDVASRGLDVEDVKFVINFDYPNNSEDYIHRIGRTARSQKTGTAYTFFTPNNMKQAHDLVSVLREANQAINPKLIQMAEDRGGKSNWSFKGRTRWRV

Ddx5 Mutant

MPGYSDRDRGRDRGSYSSGPPRFGGSRNGPPPAKKFGNPGDRLEALEPG

**Figure S2. Amino acids sequences of wild-type and mutant Ddx5 proteins.** Green letters indicate the Q motif. Blue letters indicate the DEAD box. Red letters indicate missense amino acids.

Figure S3

*ddx5* gene

ATGCCTGGATATTCTGACAGAGACCGCGGTCGTGACAGAGGTAGTTACAGCAGTGGACCACCACGTTTTGGAGGCAGCAGAAATGGACCGCCTCCAGCTAAGAAGTTTGGGAATCCAGGTGACCGTCTGCGGAAAAAGCACTGGAACCTGGATGAGCTCCCCAAGTTTGAAAAGAACTTCTACCAAGAGAATCCTGATGTTGCTCGGAGGTCAGCTCAAGAAGTTGAACACTACAGGAGAAGCAAGGAGATCACAGTTAAGGGTCGAGACGGTCCCAAACCCATCGTCAAATTTCATGAAGCCAACTTTCCAAAGTATGTGATGGATGTGATCACTAAACAGAACTGGACTGATCCAACCCCTATCCAAGCTCAGGGGTGGCCAGTTGCACTGAGTGGCAAAGATATGGTTGGCATTGCACAGACTGGATCTGGAAAAACACTCTCGTACCTGTTGCCTGCTATTGTTCACATAAACCACCAACCATTCCTGGAGCACGGAGATGGGCCCATTTGTTTGGTATTGGCTCCCACCCGTGAGTTGGCCCAGCAAGTCCAGCAGGTGGCAGCAGAGTATGGGAAAGCTTCTCGTATAAAGTCCACCTGCATCTATGGAGGTGCTCCCAAAGGACCACAGATCAGGGATCTGGAAAGGGGTGTTGAGATTTGTATTGCCACACCTGGAAGACTTATCGATTTCCTTGAAGCCGGAAAAACAAATCTGCGCAGATGCACATATCTTGTACTTGATGAAGCTGACCGGATGCTTGACATGGGATTTGAACCACAAATTCGAAAAATCGTGGACCAAATTAGGCCGGACAGACAGACACTCATGTGGAGTGCTACATGGCCCAAAGAGGTGAGGCAGCTGGCTGAGGACTTCTTGAAGGAGTATATACAGATCAATGTTGGTGCTCTGCAGCTCAGTGCCAACCACAACATCCTCCAAATTGTTGATGTTTGCAATGATGGCGAGAAAGAGGACAAACTGATCCGTCTGCTGGAGGAAATCATGAGCGAGAAGGAAAACAAGACAATTATCTTTGTGGAGACCAAAAGGAGGTGTGATGACCTTACTAGGCGGATGCGCAGGGATGGGTGGCCAGCAATGGGCATTCATGGAGACAAGAACCAGCAAGAAAGAGACTGGGTGCTCAATGAATTTAAATACGGCAAAGCGCCCATCCTAATTGCCACAGATGTCGCCTCCAGAGGACTAGATGTGGAGGACGTCAAATTTGTCATTAACTTTGACTACCCCAACAATTCTGAGGACTACATTCACCGCATTGGCCGAACAGCTCGCAGTCAGAAAACGGGCACAGCCTACACATTCTTTACGCCCAACAACATGAAACAGGCTCACGACCTCGTCTCAGTCCTCCGAGAGGCCAACCAAGCCATAAACCCCAAGCTCATCCAAATGGCTGAAGACAGAGGAGGTAAATCCAATTGGTCGTTCAAGGGGAGGACGAGGTGGAGGGTATAG

*ddx5* mutant gene

ATGCCTGGATATTCTGACAGAGACCGCGGTCGTGACAGAGGTAGTTACAGCAGTGGACCACCACGTTTTGGAGGCAGCAGAAATGGACCGCCTCCAGCTAAGAAGTTTGGGAATCCAGGTGACCGTCTGGAAGCACTGGAACCTGGATGA

**Figure S3. Nucleotide sequences of the *ddx5* gene and *ddx5* mutant gene.** Red letters and blue letters indicate start codon and stop codon, respectively.

**
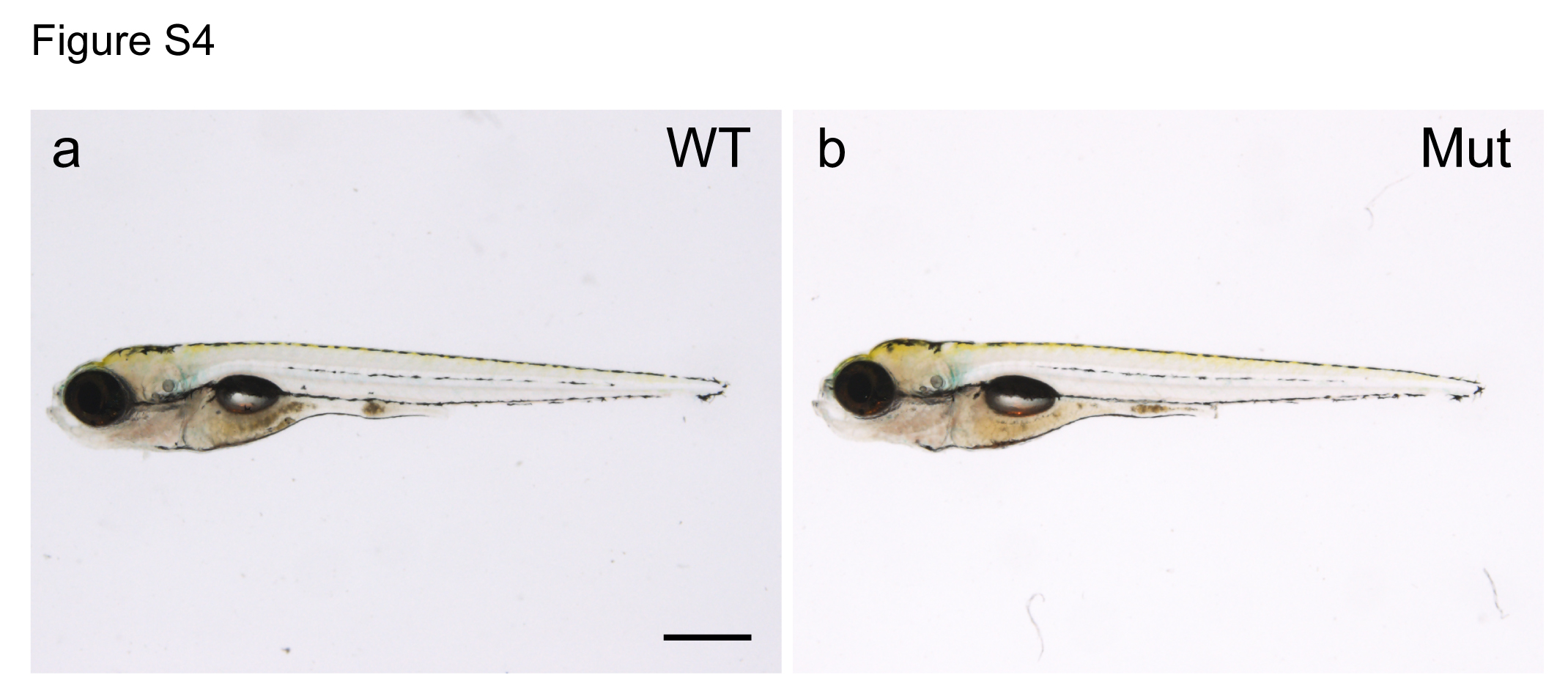
**

**Figure S4** **Morphology of *ddx5*-deficient fish at 5 dpf.**

(a) Live wild-type embryo at 5 dpf. (b) Live *ddx5*-deficient mutant embryo at 5 dpf. Scale bar, 500 μm.

**
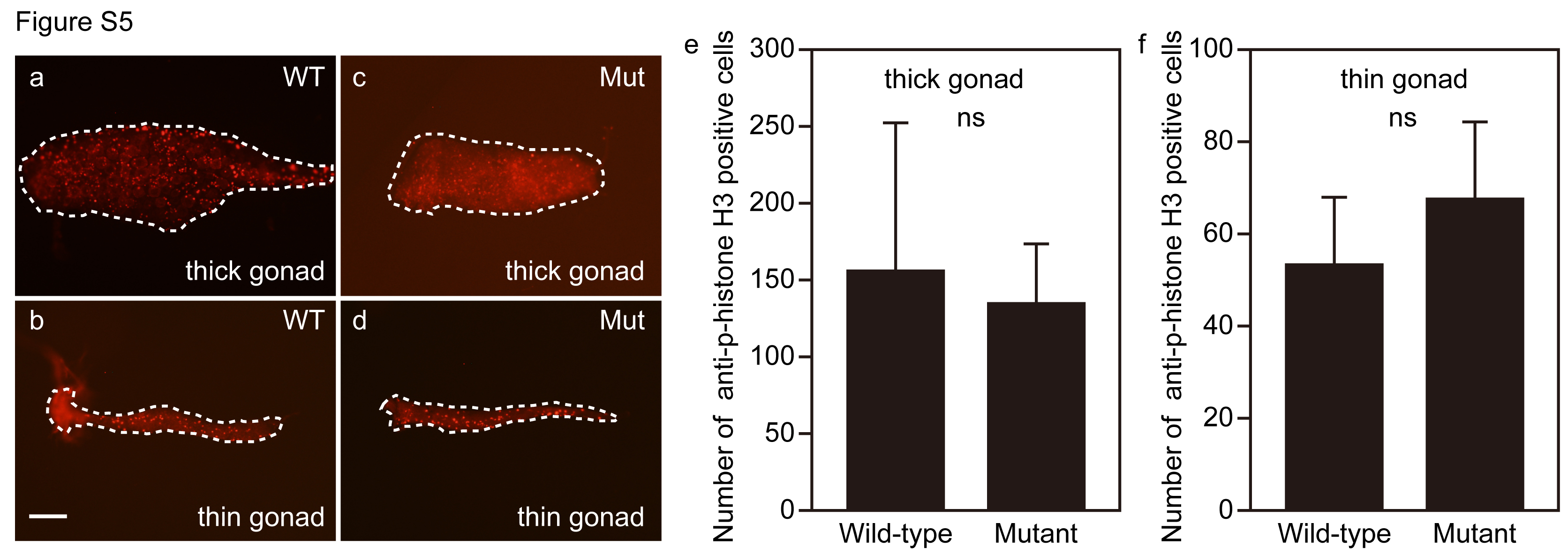
**

**Figure S5** **Proliferating cells in immature gonads of *ddx5*-deficient fish.**

(a-d) Immunostaining with anti-phospho-histone H3 antibody. (a, b) Wild-type gonads. (c, d) *ddx5*-deficient mutant gonads. Scale bar, 200 μm. (a, c) Thick immature gonads. (b, d) Thin immature gonads. Wild-type and *ddx5*-deficient mutant gonads were incubated with anti-phospho-histone H3 antibody to detect proliferating cells. (e, f) The number of proliferating cells in the wild-type (e; n=5, total 779 positive cells) (f; n=4, total 214 positive cells) and *ddx5*^-/-^ mutants (e; n=4, total 530 positive cells) (f; n=7, total 474 positive cells) was counted. Error bars indicate standard deviation. ns: not significant.

**
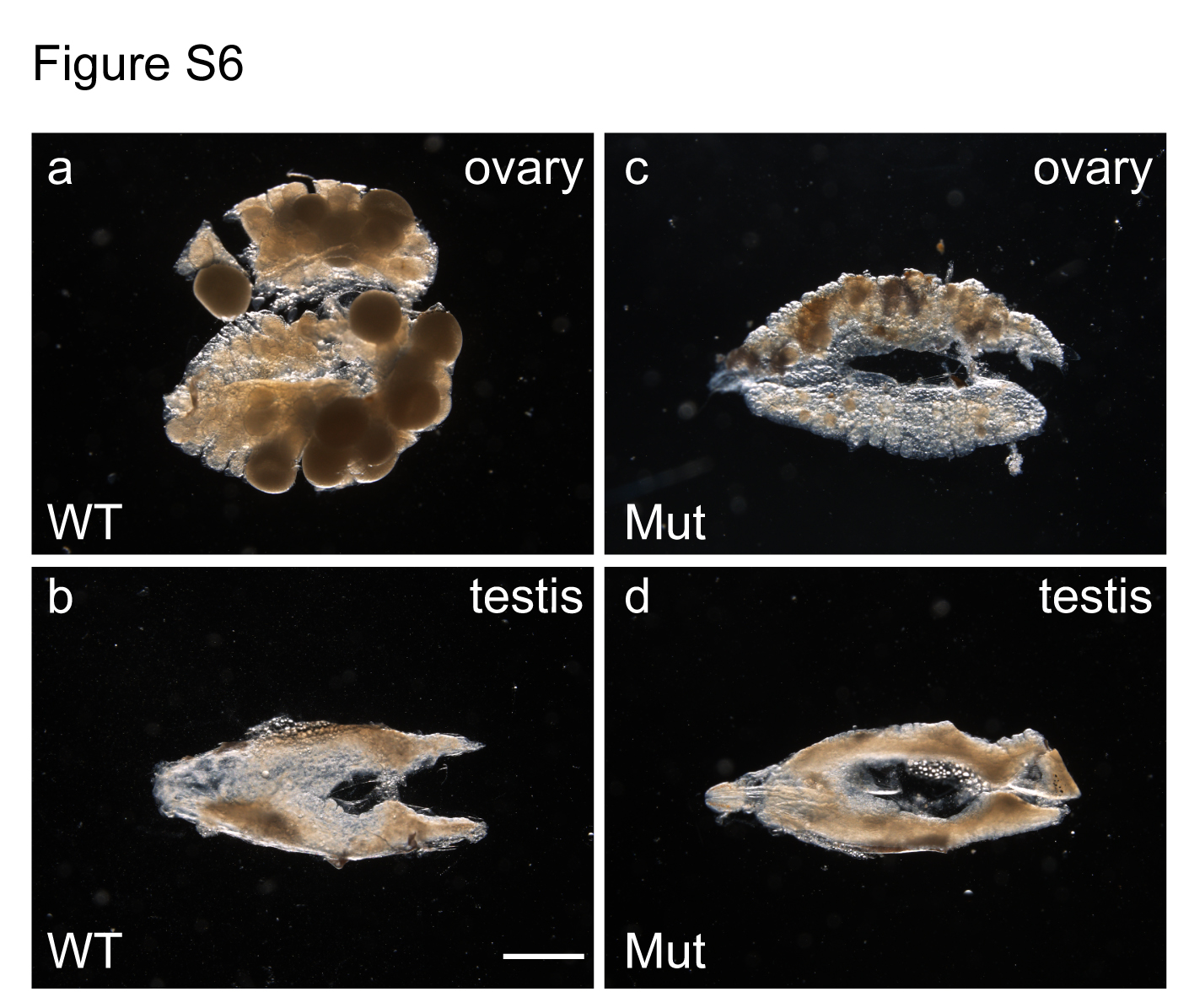
**

**Figure S6** **Morphology of *ddx5*-deficient gonads at 90 dpf.** (a, b) *ddx5^+/-^* wild-type. (c, d) The *ddx5^-/-^* mutant. The ovaries of *ddx5*-deficient females had small ovaries compared to wild-type at 90 dpf, whereas the testes of wild-type and *ddx5*-deficient fish were similar. Genotyping of individual fish was performed by genomic PCR. Scale bar, 1 mm.

**Supplemental Tables**

**Table S1 Amino acids sequence of *ddx5*-TALENs used in this study.**

| *ddx5*-TALEN-F |
| --- |
| MAPKKKRKVDYKDHDGDYKDHDIDYKDDDDKGTVDLRTLGYSQQQQEKIKPKVRSTVAQHHEALVGHGFTHAHIVALSQHPAALGTVAVTYQHIITALPEATHEDIVGVGKQWSGARALEALLTDAGELRGPPLQLDTGQLVKIAKRGGVTAMEAVHASRNALTGAPLPLNLTPDQVVAIASNHGGKQALETVQRLLPVLCQDHGLTPDQVVAIASNHGGKQALETVQRLLPVLCQDHGLTPDQVVAIASNHGGKQALETVQRLLPVLCQDHGLTPDQVVAIASNIGGKQALETVQRLLPVLCQDHGLTPDQVVAIASNIGGKQALETVQRLLPVLCQDHGLTPDQVVAIASNGGGKQALETVQRLLPVLCQDHGLTPDQVVAIASHDGGKQALETVQRLLPVLCQDHGLTPDQVVAIASHDGGKQALETVQRLLPVLCQDHGLTPDQVVAIASNIGGKQALETVQRLLPVLCQDHGLTPDQVVAIASNHGGKQALETVQRLLPVLCQDHGLTPDQVVAIASNHGGKQALETVQRLLPVLCQDHGLTPDQVVAIASNGGGKQALETVQRLLPVLCQDHGLTPDQVVAIASNHGGKQALETVQRLLPVLCQDHGLTPDQVVAIASNIGGKQALETVQRLLPVLCQDHGLTPDQVVAIASHDGGKQALESIVAQLSRPDPALAALTNDHLVALACLGGRPAMDAVKKGLPHAPELIRRVNRRIGERTSHRVAGSQLVKSELEEKKSELRHKLKYVPHEYIELIEIARNSTQDRILEMKVMEFFMKVYGYRGKHLGGSRKPDGAIYTVGSPIDYGVIVDTKAYSGGYNLPIGQADEMQDYVEENQTRDKHINPNEWWKVYPSSVTEFKFLFVSGHFKGNYKAQLTRLNHITNCNGAVLSVEELLIGGEMIKAGTLTLEEVRRKFNNGEINF |
| *ddx5*-TALEN-R |
| MAPKKKRKVYPYDVPDYAGYPYDVPDYAGSYPYDVPDYAAHGTVDLRTLGYSQQQQEKIKPKVRSTVAQHHEALVGHGFTHAHIVALSQHPAALGTVAVTYQHIITALPEATHEDIVGVGKQWSGARALEALLTDAGELRGPPLQLDTGQLVKIAKRGGVTAMEAVHASRNALTGAPLNLTPDQVVAIASHDGGKQALETVQRLLPVLCQDHGLTPDQVVAIASHDGGKQALETVQRLLPVLCQDHGLTPDQVVAIASNIGGKQALETVQRLLPVLCQDHGLTPDQVVAIASNHGGKQALETVQRLLPVLCQDHGLTPDQVVAIASNHGGKQALETVQRLLPVLCQDHGLTPDQVVAIASNGGGKQALETVQRLLPVLCQDHGLTPDQVVAIASNGGGKQALETVQRLLPVLCQDHGLTPDQVVAIASHDGGKQALETVQRLLPVLCQDHGLTPDQVVAIASHDGGKQALETVQRLLPVLCQDHGLTPDQVVAIASNIGGKQALETVQRLLPVLCQDHGLTPDQVVAIASNHGGKQALETVQRLLPVLCQDHGLTPDQVVAIASNGGGKQALETVQRLLPVLCQDHGLTPDQVVAIASNHGGKQALETVQRLLPVLCQDHGLTPDQVVAIASHDGGKQALESIVAQLSRPDPALAALTNDHLVALACLGGRPAMDAVKKGLPHAPELIRRVNRRIGERTSHRVAGSQLVKSELEEKKSELRHKLKYVPHEYIELIEIARNSTQDRILEMKVMEFFMKVYGYRGKHLGGSRKPDGAIYTVGSPIDYGVIVDTKAYSGGYNLPIGQAREMQRYVEENQTRNKHINPNEWWKVYPSSVTEFKFLFVSGHFKGNYKAQLTRLNRITNCNGAVLSVEELLIGGEMIKAGTLTLEEVRRKFNNGEINF |

Red letters indicate two variable amino acids in their RVD (repeat-variable di-residue).

**Table S2 PCR primers used in this study.**

| Primer name | Sequence (5’ to 3’) |
| --- | --- |
| ddx5-HMA-F1 | CCTCCAGCTAAGTTTGGGAAT |
| ddx5-HMA-R1 | ACATCAGGATTCTCTTGG |
